# Biomechanical Gait Analysis of an Adult with Severe Hemophilia: A Case Report

**DOI:** 10.3390/hematolrep14020017

**Published:** 2022-03-31

**Authors:** Claudiane A. Fukuchi, Alessandro R. Zorzi, Reginaldo K. Fukuchi, Janaina B. S. Ricciardi, Glenda Feldberg, Alberto Cliquet

**Affiliations:** 1 Department of Orthopedics and Traumatology, Faculty of Medical Sciences, University of Campinas-UNICAMP, São Paulo 13083-970, Brazil; arzorzi@hc.unicamp.br (A.R.Z.); cliquet@unicamp.br (A.C.J.); 2 Biomedical Engineering Program, Federal University of ABC, São Paulo 09606-070, Brazil; reginaldo.fukuchi@ufabc.edu.br; 3 Hemophilia Treatment Center (HTC) ‘Cláudio Luiz Pizzigatti Corrêa’, University of Campinas–UNICAMP, São Paulo 13083-878, Brazil; janaina_bosso@hotmail.com (J.B.S.R.); glenda.fisio@uol.com.br (G.F.); 4 Department of Electrical Engineering, University of São Paulo–USP, São Paulo 13566-590, Brazil

**Keywords:** hemophilia, gait analysis, kinematics, kinetics

## Abstract

Hemophilia is characterized by recurrent bleeding into the joints leading to irreversible chronic arthropathy with reduced joint range of motion (ROM), which may lead to changes in gait patterns. To analyze the gait pattern in a 35-year-old male with severe hemophilia A, three-dimensional biomechanical analysis was performed during overground walking. The control group data from a public gait dataset of 10 healthy male individuals were used for comparison. The clinical examination was assessed with the Functional Independence Score in Hemophilia (FISH), Haemophilia Activities List (HAL), and Hemophilia Joint Health Score (HJHS). The biomechanical analysis demonstrated a pattern for both left knee and ankle joints with greater similarity to the control group compared to the right knee and ankle joints. ROM based on the HJHS questionnaire also showed greater impairment of the right-side knee joint compared to the left-side knee joint. This unique pattern could be the result of a compensation mechanism due to limited movement during the walking task and the surgical treatment.

## 1. Introduction

Hemophilia is a congenital deficiency with a prevalence of 1 out 10,000 male births for hemophilia A, and 1 out 30,000 male births for hemophilia B [1]. Hemophilia is characterized by inflammation and cartilage destruction in the synovial joints due to the accumulation of blood [2], where the knee and ankle are the most involved joints in children and adults [3]. The consequences of recurrent bleeding into the joints lead to irreversible chronic arthropathy [4,5,6], characterized by a reduced joint range of motion, flexion contracture, and swelling, which may substantially impair the gait patterns. Currently, the standard therapy for hemophilia includes controlling or preventing bleeding with a specific coagulation factor; and surgical intervention to improve joint function when necessary [5,7].

One way to characterize the gait pattern of pathologic individuals is through a three-dimensional (3D) biomechanical analysis where a motion capture system composed of high-speed video cameras and force platforms is typically employed. This analysis technique has been widely used as a functional diagnostic tool [8] to assist clinicians in their decision-making process to optimize the care of pathological individuals [9] such as hemophilic individuals [10]. In fact, previous studies have examined the gait patterns of individuals with hemophilia; however, they have reported only spatiotemporal parameters, joint angles, or pedobarography-related variables [11,12,13]. Few studies have employed 3D gait biomechanics to describe hemophilic gait patterns and most of those that did employ this technique had the subjects walk on a treadmill during the experiments [14,15,16,17]. In reality, differences in gait biomechanics have been widely reported when comparing overground and treadmill walking in healthy individuals [18,19]; furthermore, the treadmill imposes a more challenging environment for older individuals [20]. This effect could also be extended to individuals with pathologies such as those presenting hemophilia, therefore interfering with the external validity of the previous findings. More recently, Putz et al. [13] compared the hemophilic gait pattern with healthy controls during overground level walking. However, the authors did not report lower-extremity joint moments despite the importance of these variables to describe pathological gait patterns [21,22]. Stephensen et al. also performed biomechanical gait analysis during overground level walking; however, only children with hemophilia were analyzed [23,24,25]. Moreover, only the peak values were compared between the control and hemophilic group, where a unique instant was analyzed instead of the whole instance of the gait cycle [23,24]. Therefore, given the importance of describing the hemophilic gait pattern and the lack of studies on this topic in adults, the objective of this case report was to perform and present the results of a 3D biomechanical gait analysis of a hemophilic individual during overground walking.

## 2. Case Report

**Figure 1:**
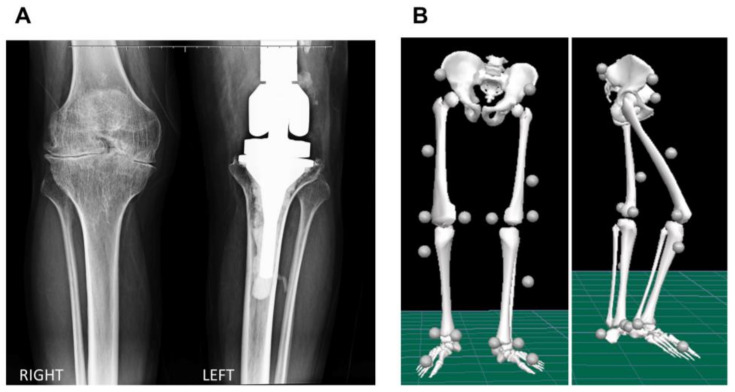
(**A**) Standing anterior–posterior radiographic view of the right and left knees, and (**B**) frontal and sagittal view of the biomechanical model displaying the marker set convention adopted in the study.

A 35-year-old male (body mass: 73 kg; height: 172 cm) with severe hemophilia A, no target joint, and an annualized joint bleeding rate of zero during the previous twelve months volunteered for this study. Prophylaxis had been initiated at the age of 26 years after osteosynthesis of the right olecranon. He was receiving rFVIII tertiary prophylaxis (13.5 UI/kg three times a week), with no inhibitor history. He has an endoprosthesis in the left knee due to an automobile accident at the age of 30 years, which presented signs of loosening. The individual currently suffers from chronic bilateral hemophilic arthropathy of elbow, knee, and ankle joints. The severity of the arthropathy in the right knee was determined with the Arnold–Hilgartner classification as stage IV based on the X-ray image (Figure 1A). Prior to participation, he read and signed a consent form (CAAE 22437419.8.0000.5404). 

### 2.1. Biomechanical Assessment

**Figure 2:**
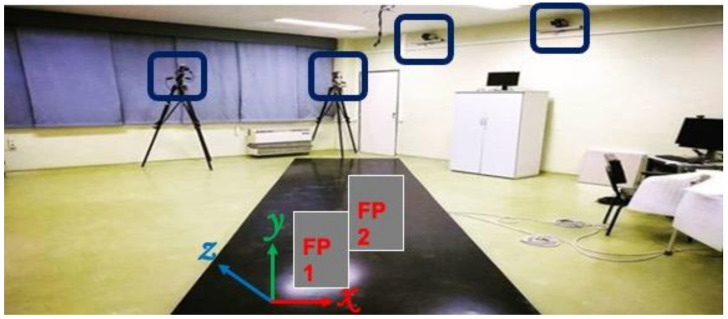
Overview of the area of the data collection showing 4 of the 12 high-speed cameras and the two force plates (force plate 1 (FP1) and force plate 2 (FP2)).

The hemophilic individual was asked to perform overground walking trials at a comfortable, self-selected speed while the trajectories of reflective markers and external forces were recorded with twelve high-speed cameras (Vero 1.3, Vicon Motion Systems Ltd., Oxford, UK) with a sampling rate of 100 Hz, and two force plates (AMTI, Watertown, MA, USA) at 1000 Hz, respectively (Figure 2). A Plug-in-Gait marker set was adopted to quantify the kinematics and kinetics variables of the knee and ankle joints during walking (Figure 1B). Knee flexion/extension and ankle dorsiflexion/plantarflexion angles and internal moments, in addition to the vertical ground reaction forces (GRF) variables, were analyzed. The data were processed and analyzed in Visual 3D software (C-Motion Inc., Germantown, MD, USA), and the kinetics data were spatially normalized by body mass. To assess the gait pattern of the hemophilic individual, the data of a public gait dataset of 10 healthy male individuals (age 29.5 ± 4.6 years, height 173.3 ± 8.0 cm, body mass 73.5 ± 8.5 kg) performing overground walking at a comfortable speed [19] was considered the control group data and used for comparison in this study. To enhance the interpretation of the findings, we provided the root mean square error (RMSE) values as a metric to compare the differences of the biomechanics variables between the hemophilic data and the control group data throughout the gait cycle. 

### 2.2. Clinical Assessment

The clinical examination was assessed by two experienced physiotherapists and the following clinical scores were applied: Functional Independence Score in Hemophilia (FISH) = 13/32, Haemophilia Activities List (HAL) = 73.16/100, and Hemophilia Joint Health Score (HJHS) = 84/124. The FISH score of 13/32 revealed that the subject presents functional limitations in eating, brushing, dressing, and, especially, in lower limb activities, such as chair transferring, squatting, and walking, and is not able to perform the functional activities of stairs and running. The HAL score of 73.16/100 reveals that, despite the subject having significant functional limitations observed by the FISH score, his functional self-perception is good. For the HJHS, specific scores were found for the elbow (right = 15 and left = 13), knee (right = 18 and left = 14), and ankle (right = 8 and left = 12) joints. The range of motion (ROM) measured with the goniometer of the specific joints that compose the HJHS score are described in Table 1. 

**Figure 3:**
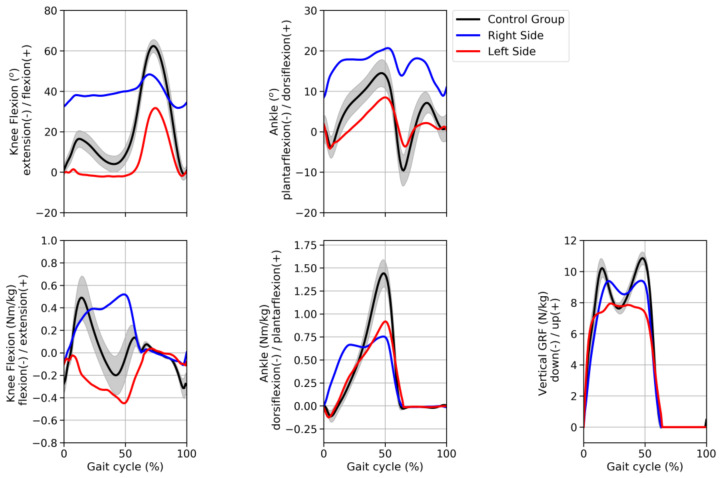
Joint angles (top graphs) and joint moments (bottom graphs) of the knee and ankle joints and GRF (bottom graph) time series curves normalized by the gait cycle. The curves of the hemophilic individual (blue and red curves) represent his average pattern whereas the curves of the control group represent the average (±1 standard deviation) of 42 subjects of a public walking biomechanics dataset.

Figure 3 displays the knee and ankle angles and moments normalized time series curves of both the hemophilic individual and the control group. The hemophilic individual showed a greater flexion pattern of the right knee, while the left knee presented a more extended pattern, particularly during the stance phase, compared with the control group. Both knees presented reduced flexion ability (range of motion) during the swing phase of the gait compared to the control group data. Overall, the left knee presented a more similar pattern, compared to the control group data, than the right knee as revealed by the RMSE 18.03° and 24.41° for the left and right knees, respectively. Similarly, the right ankle exhibited greater dorsiflexion throughout the gait cycle compared to both the left ankle and the control group patterns. Indeed, the left ankle displayed a more similar pattern when compared to the control group data, as observed by the lower value of the RMSE (left = 4.63°, right = 13.56°) value.

Knee joint moments presented contrasting patterns between sides when compared to the control group data. The right knee exhibited a greater extension moment compared to both left-side and control group data. However, the RMSE showed similar values between right (0.32 Nm/kg) and left knee moments (0.31 Nm/kg) compared to the control group data. The ankle joint presented similar patterns between right and left sides, with the right ankle presenting a reduced peak ankle plantarflexion moment compared to the left ankle; however, both right and left peaks were lower than the control group peak. RMSE results revealed that the left ankle moment was more comparable to the control group data than the right ankle moment (0.22 Nm/kg vs. 0.32 Nm/kg, respectively). Additionally, the vertical GRF showed more similar patterns in the right side (0.93 N/kg) than the left side (1.36 N/kg), when compared to the control group.

## 3. Discussion 

The aim of this study was to investigate the gait pattern of an individual with severe hemophilia A while performing level overground walking trials. Despite hemophilia being characterized by joint deterioration, which may impair the walking pattern, previous studies have solely reported the results of pedobarography [11,12], joint angles [13], or treadmill walking [14,15,16,17]. Moreover, compared with healthy male controls, patients with hemophilia presented no difference in the Gait Deviation Index score [13], where only kinematic variables were assessed. The results of the present study showed that, compared to a reference dataset (control group), the hemophilic individual presented contrasting knee kinematics and kinetics patterns as observed in the time-series curves displayed in Figure 3 and RMSE values. The biomechanical analysis showed that both knees presented reduced flexion ability during the swing phase of the gait in comparison with the control group data. A previous study had also reported this pattern during the swing phase in individuals with hemophilia but on the treadmill [16]. In contrast, increased knee flexion was found in individuals with ankle osteoarthritis secondary to hemophilia who also walked on the treadmill [15]. Based on the RMSE results, the right knee presented a more impaired pattern, compared to the control group data, than the left knee. Moreover, the HJHS score further demonstrated a more functionally compromised right knee compared to the left knee. Hence, the results of both the biomechanical and clinical assessment indicate that the right knee was more affected than the left knee. These results may be explained by the fact that the left knee had undergone a total knee arthroplasty (TKA). Additionally, the knee joint was the most compromised joint based on both biomechanics and the HJHS questionnaire, which corroborate the previous study that had also characterized the knee as the most involved joint in the HJHS questionnaire [26]. Regarding the ankle joint, the left ankle presented more similar biomechanical patterns compared to the control group, as opposed to the right side where a greater dorsiflexion pattern throughout the gait cycle was observed. The range of motion based on the HJHS score is in agreement with this finding as a greater dorsiflexion angle on the right side, compared with the left side, was observed. 

The contrasting patterns of the joint moments, which indicate the amount of joint effort during walking, between right and left knees could be a result of either kinematics or the external forces, or both, as these physical quantities are the input information required for the calculation of joint moments. In fact, the vertical GRF pattern of the right side, compared to the left, is more similar to the control group. Hence, the present findings suggest that when walking, the hemophilic individual relied more on his right knee since both the joint moments and vertical GRF pattern were higher compared with the left side. We speculate that this pattern is a compensation mechanism due to the mitigation of joint deformity given by the TKA procedure. In contrast with previous studies, which performed the walking trials either on a treadmill [15] or in pre-adolescent boys [24], our results showed higher knee flexion and lower ankle plantarflexion moments compared with the control group. One of the main drawbacks of hemophilia is decreased ROM due to the inflammation and arthropathy as a consequence of the recurrent episodes of bleeding into the joints, which can alter the gait pattern. In a point of fact, both knee and ankle joints exhibited reduced ROM throughout the gait cycle, which is in agreement with a previous study [12]. The hemophilic individual in this study had undergone a previous surgery in his left knee, which seems to have positively influenced his gait patterns as the RMSE values were lower in the left compared to the right knee and ankle joints; furthermore, the results were in contrast with previous studies [15,24]. Conversely, the pattern of the right side seems to have been adopted to compensate for the limited movement and pain, especially in the knee joint. Additionally, whereas the gait speed for the control group was 1.19 m/s, for the hemophilic individual the gait speed was 1.10 m/s. However, this gait speed (1.10 m/s) was within one standard deviation (± 0.13 m/s) of the mean speed adopted by the control group. Despite not being certain whether speed played a major role in explaining the present results, gait speed should always be controlled when comparing able-bodied individuals with pathological individuals, as we know that gait speed might affect the biomechanical variables [27].

There are some limitations in this study that must be highlighted. These results should not be employed to characterize the hemophilic gait pattern since a single individual was analyzed. Additionally, although the conduction of gait analysis and the interpretation of the results may be challenging due to the complex nature of the process involved in measuring human movement and the higher number of biomechanical variables [28], the use of gait indices such as the Gait Profile Score [29] and Gait Deviation Index [30] have been proposed to enable the assessment of the overall quality movement pattern based on a single number. Thus, these gait indices could easily be applied, facilitating the interpretation of the gait analysis in routine clinical care. As this pathology is known to affect multiple joints, future studies with a greater sample size applying complex statistical analyses or gait indices are necessary to tackle these issues. The use of gait analysis instrumentation in this study was paramount to capture the impairments in the walking patterns of the hemophilic individual compared to a normative dataset of healthy controls. Therefore, routine use of this technique within a clinical context may help clinicians in their decision-making process of selecting the optimal intervention to target specific movement impairments.

## 4. Conclusions

The kinematics and kinetics patterns of the left knee and left ankle joints presented more similar patterns than the right side compared to the reference gait data. This unique pattern may be a result of a compensation mechanism and the surgical treatment (i.e., TKA); this hypothesis, however, requires further investigation in future studies. Taken together, these results indicate that biomechanical gait analysis appears to be useful in detecting walking alterations as a consequence of hemophilia progression with the advantage of being a non-invasive procedure. 

## Figures and Tables

**Table 1:** Range of motion of the flexion and extension movement of the elbow, knee, and ankle joints.

	Elbow		Knee		Ankle	
	Left	Right	Left	Right	Left	Right
**Flexion (°)**	134	112	78	60	36	46
**Extension (°)**	−32	−45	0	−36	13	−2

## Data Availability

The data presented in this study is available on request from the corresponding author. The dataset analyzed is available in Figshare repository, https://doi.org/10.6084/m9.figshare.5722711.v4.
